# Structure, function, and plasticity of GABA transporters

**DOI:** 10.3389/fncel.2014.00161

**Published:** 2014-06-17

**Authors:** Annalisa Scimemi

**Affiliations:** Department of Biology, SUNY AlbanyAlbany, NY, USA

**Keywords:** GABA, GABA transporters, uptake, synaptic transmission, synaptic plasticity, GAT1, GAT3, SLC6

## Abstract

GABA transporters belong to a large family of neurotransmitter:sodium symporters. They are widely expressed throughout the brain, with different levels of expression in different brain regions. GABA transporters are present in neurons and in astrocytes and their activity is crucial to regulate the extracellular concentration of GABA under basal conditions and during ongoing synaptic events. Numerous efforts have been devoted to determine the structural and functional properties of GABA transporters. There is also evidence that the expression of GABA transporters on the cell membrane and their lateral mobility can be modulated by different intracellular signaling cascades. The strength of individual synaptic contacts and the activity of entire neuronal networks may be finely tuned by altering the density, distribution and diffusion rate of GABA transporters within the cell membrane. These findings are intriguing because they suggest the existence of complex regulatory systems that control the plasticity of GABAergic transmission in the brain. Here we review the current knowledge on the structural and functional properties of GABA transporters and highlight the molecular mechanisms that alter the expression and mobility of GABA transporters at central synapses.

## Introduction

The brain utilizes GABAergic synaptic transmission to modulate the ongoing activity of neuronal networks. By acting on ionotropic and metabotropic receptors, GABA controls the generation of membrane potential oscillations, the time window over which synaptic inputs are integrated and the temporal structure of the activity patterns produced by entire populations of neurons (Pouille and Scanziani, 2001; Hajos et al., 2004; Akam and Kullmann, 2010; Mann and Mody, 2010; Stark et al., 2013). These actions require a fine control of the timing of GABA receptor activation which, in turn, depends on the precise timing of GABA release from pre-synaptic terminals and GABA clearance from the extracellular space. Extracellular GABA is not subject to enzymatic breakdown, and its clearance relies entirely on diffusion and uptake by specific transporters. GABA transporters belong to a large family of neurotransmitter:sodium symporters, and are widely expressed throughout the brain. GABA transporters are expressed in different cell types, including neurons and astrocytes, at expression levels that vary across different brain regions. Experimental evidence indicates that the distribution of GABA transporters in the cell membrane is highly dynamic and can be modified in an activity-dependent manner. For example, there are intracellular signaling cascades that regulate the cytoplasm-to-surface partitioning of GABA transporters (Corey et al., 1994; Whitworth and Quick, 2001) and the interaction between GABA transporters and components of the cytoskeleton, which control the mobility of these molecules within the cell membrane (Imoukhuede et al., 2009). In this review, we provide an overview of the structural and functional properties of GABA transporters and of the molecular mechanisms that can alter their expression and mobility in the cell membrane.

## The GABA transporter family and the GABA transporter group

**Figure 1:**
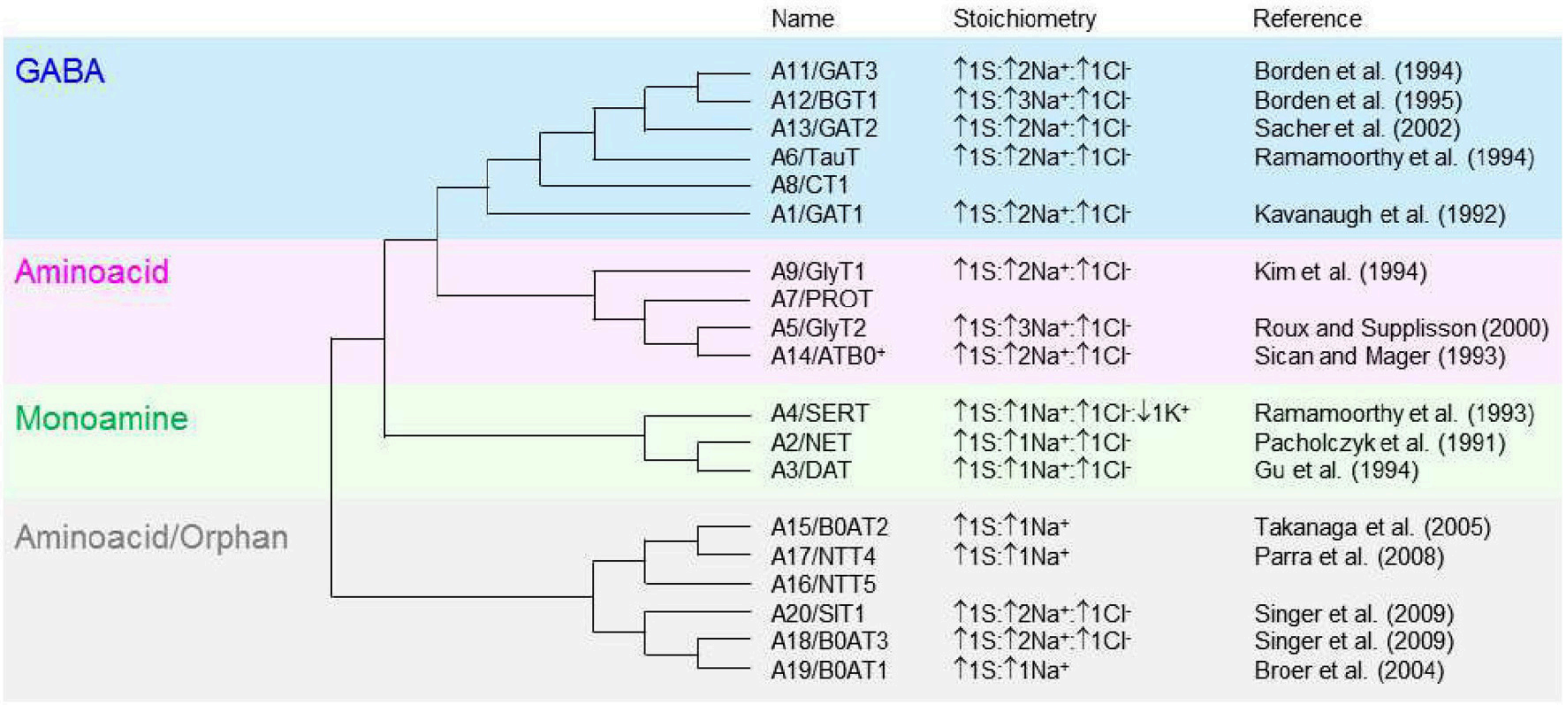
**Molecular phylogenetic analysis of the SLC6 neurotransmitter transporter family in *Homo sapiens***. The SLC6 family is divided into four groups, including the GABA (blue), aminoacid (pink), monoamine (green) and aminoacid/orphan transporters (gray). The evolutionary history is inferred by using the maximum likelihood method based on the JTT matrix-based model (Jones et al., 1992). The initial tree for the heuristic search is obtained automatically by applying neighbor-join and BioNJ algorithms to a matrix of pairwise distances estimated using a JTT model, and then selecting the topology with superior log likelihood value. The analysis is performed on 19 aminoacid sequences because the gene encoding the SLC6A10 transporter is thought to be a pseudogene (Kristensen et al., 2011). All positions containing gaps and missing data were eliminated. There is a total of 372 positions in the final dataset. The evolutionary analysis is obtained with MEGA5 (Tamura et al., 2011). The tree is not drawn to scale; it includes the SLC and the commonly used neurotransmitter transporter nomenclature. The stoichiometry and direction of the transport cycle are included, when known, together with the corresponding reference.

GABA transporters belong to a family of neurotransmitter:sodium symporters that in humans is referred to as the solute carrier 6 (SLC6) family. The SLC6 family is composed of 20 members and, based on sequence composition, it is subdivided into four groups including GABA, osmolyte and creatine transporters (Figure 1, *blue section*), neurotransmitter amino acid (Figure 1, *pink section*), monoamine (Figure 1, *green section*) and nutrient amino acid/orphan transporters (Figure 1, *gray section*). The currently accepted nomenclature that is used to identify members of the SLC6 family represents the nomenclature of GABA transporters in humans (A1-20) and rats (GAT, BGT, NTT, etc.). The nomenclature of GABA transporters in mice is different and somewhat confusing when compared to the ones mentioned above. In mice, GAT2 corresponds to A12/BGT1, GAT3 corresponds to A13/GAT2 and GAT4 corresponds to A11/GAT3, while GAT1 carries the same name as in humans and rats (A1) (Nelson, 1998; Cohen-Kfir et al., 2005). In this review, for simplicity, we use the human/rat GABA transporter nomenclature. There is overlapping substrate specificity across all four groups of transporters within the SLC6 family. For example, betaine is transported by A12/BGT1 and A20/SIT1, and A9/GlyT1, A5/GlyT2, A19/B^0^AT1, A18/B^0^AT3, A14/ATB^0, +^ all transport glycine across the cell membrane (Broer and Gether, 2012). There is also overlapping substrate specificity within each group of the SLC6 family. For example, the monoamine transporters have low selectivity for decarboxylated derivatives of aromatic aminoacids like tyrosine and triptophane. Accordingly, the dopamine transporter A3/DAT also transports noradrenaline (Broer and Gether, 2012), the noradrenaline transporter A2/NET also transports dopamine (Gether et al., 2006) and the serotonin transporter A4/SERT also transports dopamine, albeit with low-affinity (Larsen et al., 2011).

The GABA transporters group comprises six transporters: A1/GAT1, A13/GAT2, A11/GAT3, A12/BGT1, A8/CT1, and A6/TauT. All members can transport GABA and other molecules and there is overlapping substrate specificity also within the GABA transporters group. For example, GAT2 and GAT3 accept substrates with a carboxyl group in the β-position and an amino group in the γ-position of their carbon backbone structure, like GABA and β-alanine. Conversely, taurine, which has a sulphonate—not a carboxyl—group in the β-position, is only transported by TauT; creatine, in which the amino group in the γ-position is part of the guanidino group, is only transported by CT1; betaine, in which this group is methylated, is only transported by BGT1. GAT1, GAT2, and GAT3 (particularly GAT1) are the most extensively characterized GABA transporters and will be the main focus of discussion in this review.

## The biophysical properties

The early experiments involving solubilization of GABA transporters from rat brains and subsequent reconstitution in proteoliposomes revealed four important biophysical properties of GABA transporters (Kanner, 1978). *First*, they showed that GABA transport is an active process, not directly coupled to ATP hydrolysis but requiring the presence of an inward electrochemical gradient for Na^+^, typically created by the membrane Na^+^/K^+^ ATPase. *Second*, GABA transporters have low micromolar, steady-state affinity for GABA, with a Michaelis-Menten constant of 2.5 μM [later found to be 3.1–10.6 μM for GAT1 (Guastella et al., 1990)]. *Third*, replacement experiments in which Li^+^, NH_4+_, Tris^+^, K^+^ were used as substitutes for Na^+^ showed that GABA transport is only supported by Na^+^, not by any of the other cations (see also Iversen and Neal, 1968). *Fourth*, the increase in GABA uptake observed in the presence of the K^+^-selective ionophore valinomycin indicated that GABA transport is electrogenic (and voltage-dependent) (Kanner, 1978). These fundamental aspects of GABA transporter function were all confirmed when GABA transporters were purified (Radian and Kanner, 1985) and identified (Radian et al., 1986) and when the first member of the GABA transporter subgroup, GAT1, was cloned (Guastella et al., 1990). Kinetic and thermodynamic experiments indicate that, in addition to Na^+^, extracellular Cl^−^ is also required for GABA transport. These findings are thought to be consistent with the existence of a co-transport mechanism for GABA, Na^+^ and Cl^−^ by GABA transporters. Accordingly, the most accredited stoichiometry of mammalian GABA transporters is 1GABA:2Na^+^:1Cl^−^. Since GABA is a zwitterionic molecule, this stoichiometry leads to a net influx of one positive charge per transport cycle (Radian and Kanner, 1983; Kavanaugh et al., 1992; Mager et al., 1993; Lu and Hilgemann, 1999b). In some radioactive tracer flux experiments, however, a net charge influx of two positive charges per transport cycle has been measured (Pastuszko et al., 1982; Loo et al., 2000). Because activation of GABA transporters triggered simultaneous influx and efflux of Cl^−^ (Loo et al., 2000), it was proposed that there is an exchange mechanism that couples Cl^−^ influx and efflux across the cell membrane. If this were the case, the proposed stoichiometry of 1GABA:2Na^+^:1Cl^−^ would only reflect the stoichiometry of GABA influx, but the net stoichiometry for the entire transport cycle would be 1GABA:2Na^+^. It has been reasoned that co-transporting Cl^−^ together with Na^+^ and GABA would add very little energy to drive the uphill transport of GABA, because the reversal potential for Cl^−^ is close to the resting membrane potential (Loo et al., 2000). This reasoning obviously holds only if considering GABA transport in cellular or sub-cellular compartments where the reversal potential for Cl^−^ is hyperpolarized, at a time when the cell is not experiencing any depolarizing event (e.g., action potential firing, sub-threshold depolarization). The counter argument, however, is that the functional relevance of an electroneutral exchange of Cl^−^ across the cell membrane is unclear. Recent kinetics experiments indicate that Cl^−^ is only required for the generation of GABA-induced steady-state currents, not for GABA-induced pre-steady-state currents, which reflect the first electrogenic steps in the transport cycle (Bicho and Grewer, 2005). This result could be explained if Cl^−^ exchange occurred in a reaction step distinct from—but thermodynamically coupled to—the translocation of GABA and Na^+^ across the membrane. The Cl^−^ exchange would occur while the transporter is in a conformational state that allows it to take up GABA and Na^+^ (Bicho and Grewer, 2005). Cl^−^ binding to the extracellular side of GAT1 could facilitate intracellular GABA release from the transporter. Therefore the entire forward transport cycle would include rapid binding and translocation of GABA and Na^+^, slower (12 ms) intracellular dissociation of GABA and Na^+^ and rapid Cl^−^ exchange (Bicho and Grewer, 2005).

## The turnover rate of GABA transporters

There are numerous and different estimates for the translocation rate of GABA and co-transported ions across the membrane via GAT1. For the forward mode (i.e., the mode that describes removal of GABA from the extracellular space toward the cytoplasm and transporter re-orientation in the cell membrane), the initial estimates at 22°C suggested a value of 2.5 s^−1^ in *Xenopus* oocytes (Radian et al., 1986). This value agrees well with the turnover rate found by others in the same preparation and in similar experimental conditions: 5.8–7.6 s^−1^ at −60 mV (Eckstein-Ludwig et al., 1999), 6.3 s^−1^ at −60 mV (Liu et al., 1998), 6–13 s^−1^ at −80 mV (Mager et al., 1993), 13 s^−1^ at −40 mV (Bicho and Grewer, 2005). However, other reports have also estimated turnover rates at 37°C and −50–90 mV of 73–93 s^−1^, much higher than it would be predicted by correcting the previous values for the estimated *Q*_10_ value of 2.8 (Gonzales et al., 2007). This discrepancy may be attributed to methodological differences, as the latter estimates are obtained using correlative freeze-fracture and electrophysiology experiments. For the reverse mode (i.e., the mode that describes GABA release into the extracellular space and transporter re-orientation in the cell membrane), the available estimates suggest turnover rates of 3 s^−1^ at −120 mV and 60 s^−1^ at +120 mV at 33°C (Lu and Hilgemann, 1999a).

## The currents associated with GABA transport

The initial biophysical characterization of GAT1 expressed heterologously in *Xenopus* oocytes did not provide an indication that GAT1 could generate any other current than the stoichiometric current described above (Hilgemann and Lu, 1999; Lu and Hilgemann, 1999a,b). Once GAT1 was expressed in HEK293 and HeLa cells, however, it became evident that GABA binding to GAT1 gates at least two more currents that are stoichiometrically uncoupled from the translocation of GABA, Na^+^ and Cl^−^ across the membrane. The two stoichiometrically uncoupled currents are: (1) an agonist-induced Na^+^ inward current (Risso et al., 1996); (2) an agonist-independent leak cationic current carried by alkali ions (Cammack and Schwartz, 1996). The lack of these currents in *Xenopus* oocytes may reflect different functional properties of GAT1 in *Xenopus* vs. mammalian expression systems (Lu and Hilgemann, 1999b). They may also be due to a technical limitation of *Xenopus* oocytes, where small Na^+^ currents are not easily resolved (Lu and Hilgemann, 1999b). Even though a fully detailed, direct comparison between the agonist-induced stoichiometrically coupled and uncoupled currents in these different expression systems is not available, the Na^+^-inward current could actually be significantly larger than one would expect based on the *Xenopus* oocytes studies. In fact, it has been suggested that this current could contribute 4-10 times more current than the stoichiometric component of GAT1 (Eckstein-Ludwig et al., 1999). Competitive GAT1 antagonists like tiagabine block both the stoichiometric and the Na^+^ inward current with *K_i_* values of 2 and 0.3 μM, respectively (Eckstein-Ludwig et al., 1999). In contrast, the GAT1 inhibitor SKF899A can be used to separate these two current components, because SKF89976A acts as a low-affinity, competitive antagonist of the stoichiometric current (*K_i_* = 7 μM) and as a high-affinity, non-competitive antagonist for the Na^+^ inward current [*K_i_* = 0.03 μM (Krause and Schwarz, 2005)]. The contribution of the leak current to the total current generated by GAT1 is modest. This is in part due to the fact that its conductance is small [*g_leak_* = 0.36 ± 0.18 nS (Cammack and Schwartz, 1996)] and in part to the fact that this current is inhibited by intracellular Na^+^ concentrations that are typically found in the cytosol of living cells (*K_i_* = 3 mM) and often reproduced during electrophysiological patch-clamp recordings (Macaulay et al., 2002). The ability of GAT1 to act as a channel as well as a transporter is not an uncommon feature among neurotransmitter transporters: it is reminiscent of the mechanisms of action of glutamate transporters, which also generate agonist-induced stoichiometrically coupled and uncoupled currents (Wadiche et al., 1995). For both GABA and glutamate transporters, the stoichiometric current is inwardly directed. However, the agonist-induced stoichiometrically uncoupled current is cationic (and depolarizing) in GABA transporters and anionic (and often hyperpolarizing) in glutamate transporters. Activation of the stoichiometrically uncoupled, glutamate transporter anionic current in retinal rod bipolar cell terminals hyperpolarizes the cell membrane and inhibits neurotransmitter release (Veruki et al., 2006). The role of the stoichiometrically uncoupled, GABA transporter cationic current is not known but one hypothesis is that activation of this current at pre-synaptic inhibitory terminals could serve as a negative feedback mechanism that, by depolarizing the cell membrane potential, ultimately inhibits GABA uptake.

One other functional aspect of the agonist-induced stoichiometrically coupled and uncoupled currents that remains currently unknown is whether they share a common permeation pathway with the substrate or if, as proposed for analogous currents generated by agonist binding to glutamate transporters, the two pathways are independent from one another (Ryan et al., 2004). Although this and other mechanistic questions about GABA transporters remain currently unanswered, the recent discovery of the crystal structure of LeuT_Aa_, a bacterial homolog from *Aquifex aeolicus* (Yamashita et al., 2005) could allow for an unprecedented detailed level of understanding of this and other details about the transport process.

## The structure of a prokaryotic GABA transporter homolog

**Figure 2:**
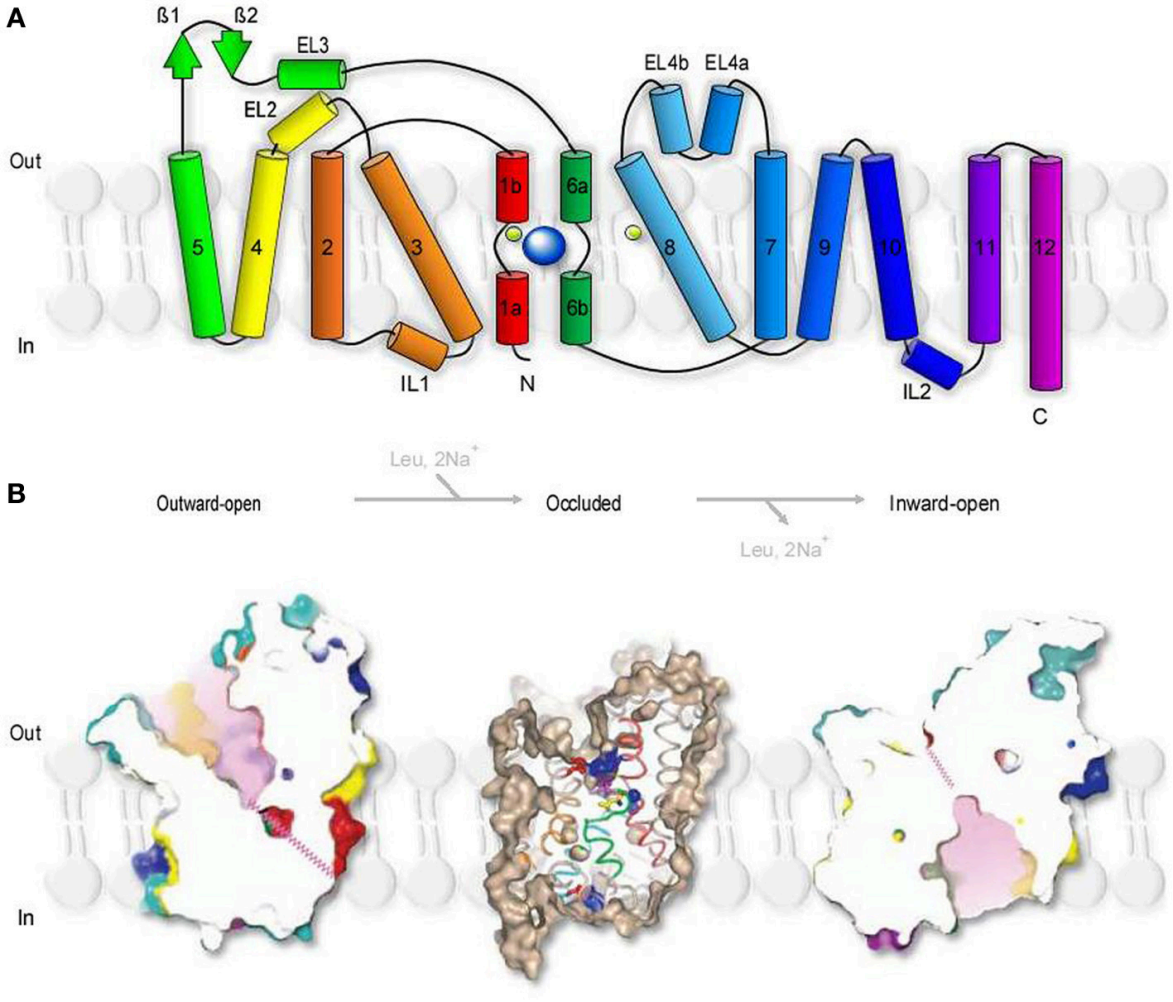
**Secondary structure and surface representation of LeuT_Aa_. (A)** Topology of *Aquifex aeolicus* LeuT_Aa_. The transporter is composed of 12 trans-membrane regions (TM1-12), with cytoplasmic N- and C-terminal domains. TM1 and TM6 are oriented antiparallel to one another and have breaks in their helical structure approximately halfway across the membrane bilayer. The transporter has two extracellular β-strands (green arrows), four extracellular (EL2, 3, 4a, 4b) and two intracellular helices (IL1, 2). The co-transported Na^+^ are depicted as two light green spheres. The substrate molecule (Leu), is depicted as a bigger blue sphere that binds to unwound regions in TM1 and TM6. Modified from (Yamashita et al., 2005). **(B)** Slice through the surface representation of LeuT_Aa_ in the Leu-free, Na^+^-bound outward-open conformation (left), in the occluded conformation where the Leu- and Na^+^-binding sites are occluded from solution in the extracellular and cytoplasmic sides (middle) and in the inward-open conformation (right). The zig-zag pink lines indicate closed intracellular pathways. Modified from (Yamashita et al., 2005) and (Krishnamurthy and Gouaux, 2012).

*Aquifex aeolicus* is a thermophilic bacterium that grows best at 95°C. The name refers to the fact that this bacterium produces water as a byproduct of its respiration (hence *Aquifex*) and was first isolated near underwater volcanic vents in the Aeolic Islands, north of Sicily (hence *aeolicus*). LeuT_Aa_ is an experimentally tractable prokaryotic leucine transporter. It is evolutionary distant from GAT1 and shares 20–25% sequence similarity with GAT1 and other members of the SLC6 family including glycine, dopamine and serotonin transporters (Yamashita et al., 2005). The proposed stoichiometry of LeuT_Aa_ (1 leucine:2 Na^+^) indicates that substrate transport via LeuT_Aa_ is not Cl^−^-dependent, in contrast to GAT1. Despite these differences, the structural conservation between LeuT_Aa_ and other members of the SLC6 family is thought to be remarkably high (Abramson and Wright, 2009). This supports the usefulness and validity of LeuT_Aa_ as a template model for the analysis of the structural architecture and function of transporters in the SLC6 family (Kristensen et al., 2011). LeuT_Aa_ has a 70Å tall and 48Å wide cylindrical structure with 12 transmembrane domains (TM1-12), intracellular N- and C-terminal domains and extracellular, glycosylated regions (Figure 2), as previously deduced from the hydropathy analysis of the aminoacidic sequence of GAT1 (Guastella et al., 1990). LeuT_Aa_ assembles as a dimer, with each protomer capable of independently binding and translocating leucine and the two co-transported Na^+^ ions. According to FRET experiments, GAT1 also assembles as a multimeric structure (Schmid et al., 2001; Moss et al., 2009). Each monomer is capable of transporting GABA independently (Soragna et al., 2005), but the multimerization process allows trafficking of GAT1 from the endoplasmic reticulum to the plasma membrane (Farhan et al., 2006). The essential core structure of LeuT_Aa_ is formed by TM1-10. TM11-12 participate in multimerization and the N- and C-terminal domains are not required for GABA transporter activity (Mabjeesh and Kanner, 1992). Consistent with these findings, other functional bacterial homologs of GAT1, like the transporter protein encoded by the tnaT gene of *Symbiobacterium thermophilum*, consist only of TM1-10 (Androutsellis-Theotokis et al., 2003). Some of the key residues in TM1-10 include: (1) Arg 69 (TM1), essential for substrate transport (Pantanowitz et al., 1993); (2) Gly80 (TM2), necessary for conformational transitions during the transport process (Zhou and Kanner, 2005); (3) Tyr140 (TM3), involved in substrate recognition and transport (Bismuth et al., 1997). One of the major novel findings recently emerged from the crystal structure of LeuT_Aa_ is that there is an internal structural repeat that allows an ideal superimposition of TM1-5 with TM6-10 by a 176.5° rotation around a pseudo-two-fold axis located in the plane of the membrane (Yamashita et al., 2005). TM1 and TM6 are oriented antiparallel to each another, with breaks in their helical structure half-way through the cell membrane. In the substrate-bound, outward-occluded and competitive inhibitor-bound outward-facing conformation, Val23 and Gly24 in TM1 and the aminoacidic residues between Ser256 and Gly260 in TM6 have extended non-helical conformations, which expose atoms that can be used for hydrogen bonding and ion coordination. The regions surrounding these unwound breaks in TM1 and TM6, together with other regions in TM3 and TM8, comprise the substrate and Na^+^-binding sites (Yamashita et al., 2005). Hinge movements allow TM1b, TM2a, TM6a to pivot around Val23, Gly55, and Leu257 and move outwards, in the Na^+^-bound, substrate-unbound outward open conformation. These movements cause the extracellular loop 3 (EL3) and TM11 to be displaced by 2.8Å and 2.2Å, respectively (Krishnamurthy and Gouaux, 2012). In the inward-open conformation, TM1b and TM6a move and block the extracellular pathway, while TM1a is tilted by 45° with respect to its position in the closed state (Krishnamurthy and Gouaux, 2012). TM2, TM5, and TM7, domains that buttress TM1 and TM6, bend and cause EL4 to move down into the extracellular vestibule and close the extracellular solvent pathway. These structural rearrangements of LeuT_Aa_ are consistent with an alternating access transport mechanism, whereby conformational changes in the structure of a transporter switch the accessibility of the substrate-binding site from the extracellular and cytoplasmic side of the membrane (Jardetzky, 1966; Lauger et al., 1980). The probability of a transporter of being in one state vs. the other depends on the energetic barriers associated with each conformational transition, which in turn depend on the substrate and co-transported ion concentration and on the membrane potential (Chung and Eaton, 2013; Schuler and Clarke, 2013).

## The distribution of GABA transporters in the brain

**Figure 3:**
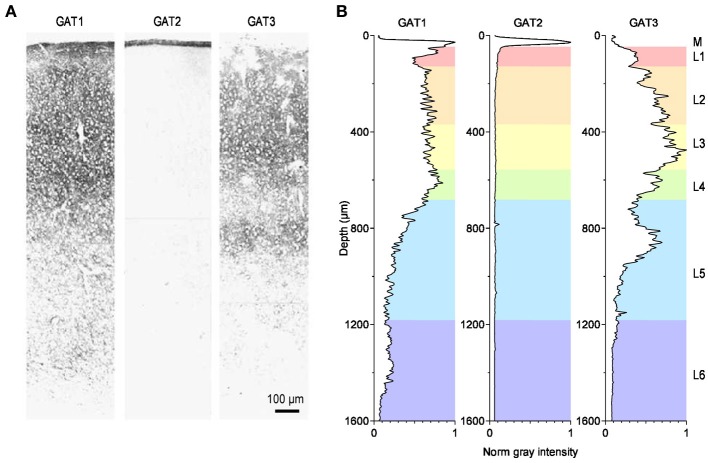
**Distribution of GABA transporters in the rat somato-sensory cortex. (A)** Immunohistochemical labeling for the GABA transporters GAT1 (left), GAT2 (middle) and GAT3 (right) in the primary somato-sensory cortex of the adult rat. Modified from (Conti et al., 2004). **(B)** Image analysis of the immunohistochemical labeling for GAT1-3. The diagrams provide a measure of the normalized, average gray value distribution measured over the entire area of the images shown in panel A. The data are normalized by the maximum gray value measured in each image. Therefore, the darkest areas, with the most intense labeling, have a normalized gray intensity value of 1. The letters on the right hand side of the figure indicate the meningeal (M) and the six cortical layers (L1–6). The gray value analysis was done using the Fiji image processing package (Schindelin et al., 2012). The rest of the analysis was performed using custom-made routines written in Igor Pro (Wavemetrics).

*In situ* hybridization studies have shown that the mRNA encoding GABA transporters is widely distributed throughout the entire central nervous system (Durkin et al., 1995; Borden, 1996). Intense labeling for the GABA transporter GAT1 is found in the cerebellum (molecular layer), basal ganglia (ventral pallidum, globus pallidus), olfactory bulb (glomerular layer), retina (inner nuclear layer), and interpeduncular nucleus. Moderate labeling is found throughout the neocortex (hippocampus proper and dentate gyrus), amygdala, septum, thalamus (ventral lateral geniculate, reticular nuclei), zona incerta, subthalamic nucleus, hypothalamus (suprachiasmatic and periventricular nuclei, anterior hypothalamic and pre-optic areas), superior colliculus, dorsal tegmental nuclei, basal ganglia (substantia nigra), nucleus of Darkschewitsch, pons and medulla (trapezoid, medial and lateral vestibular, dorsal cochlear and parabrachial nuclei, nucleus of the solitary tract and of the trigeminal nerve) and also in the spinal cord (dorsal horn laminae 1, 2, 4, 10). Weak labeling is found in cerebellar Purkinje cells, deep cerebellar nuclei and also in the spinal cord (ventral horn). In contrast to GAT1, the mRNA encoding the GABA transporter GAT2 is only detected in the leptomeninges, possibly suggesting a role for GAT2 in regulating the GABA concentration or the osmotic pressure in the cerebrospinal fluid. The mRNA encoding the GABA transporter GAT3 is less abundantly expressed than the one for GAT1. Intense labeling is found in the olfactory bulb (glomerular layer) and retina (inner nuclear layer). Moderate labeling is found in the septum (medial nucleus and vertical nucleus of the diagonal band), basal ganglia (ventral pallidum and globus pallidus), subfornical organ, amydala, thalamus (paraventricular nucleus, lateral habenula), superior colliculus, ventral tegmental nucleus, basal ganglia (substantia nigra pars compacta), and medial vestibular nucleus. Weak labeling is found in lateral reticular and parabrachial nuclei, deep cerebellar nuclei, spinal cord and in the entire neocortex (Borden, 1996). The immunohistochemical analysis largely confirms these results, particularly the faint expression of GAT2 throughout the central nervous system, as opposed to other peripheral organs (Ikegaki et al., 1994), and the widespread distribution of GAT1 and GAT3 throughout the brain (Ikegaki et al., 1994). The highest levels of expression of GAT1 are found in the hippocampus, olfactory bulb, cortical layer 1 (L1), piriform cortex, superior colliculus, interpeduncular nucleus and nucleus spinal tract of the trigeminal nerve. The highest levels of expression of GAT3 are found in the olfactory bulb, thalamus, hypothalamus, pons and medulla, globus pallidus, basal ganglia (substantia nigra), deep cerebellar nuclei, and nucleus spinal tract of the trigeminal nerve (Ikegaki et al., 1994). The density of expression of GAT1-3 in the cortex varies across layers (Figure 3). GAT1 immunostaining is highest in L2-L4, GAT2 is confined to the meninges and GAT3 is most highly expressed in L3 and upper L5 (Minelli et al., 1996; Conti et al., 2004).

## The cellular and sub-cellular distribution of GABA transporters

One of the most interesting features of GAT1 and GAT3 is their cellular and sub-cellular distribution.

In rodents, the punctate immunostaining for GAT3 is localized exclusively in astrocytic processes scattered throughout the neuropil and adjacent to symmetric and asymmetric synapses close to cell bodies, basal and apical dendrites (Minelli et al., 1996; Ribak et al., 1996; Melone et al., 2005 but see Pow et al., 2005). In the brain of other mammalian species, like cats, monkeys and humans, the astrocyte-specific expression of GAT3 is lost, because here GAT3 is also expressed in oligodendrocytes (Pow et al., 2005).

**Figure 4:**
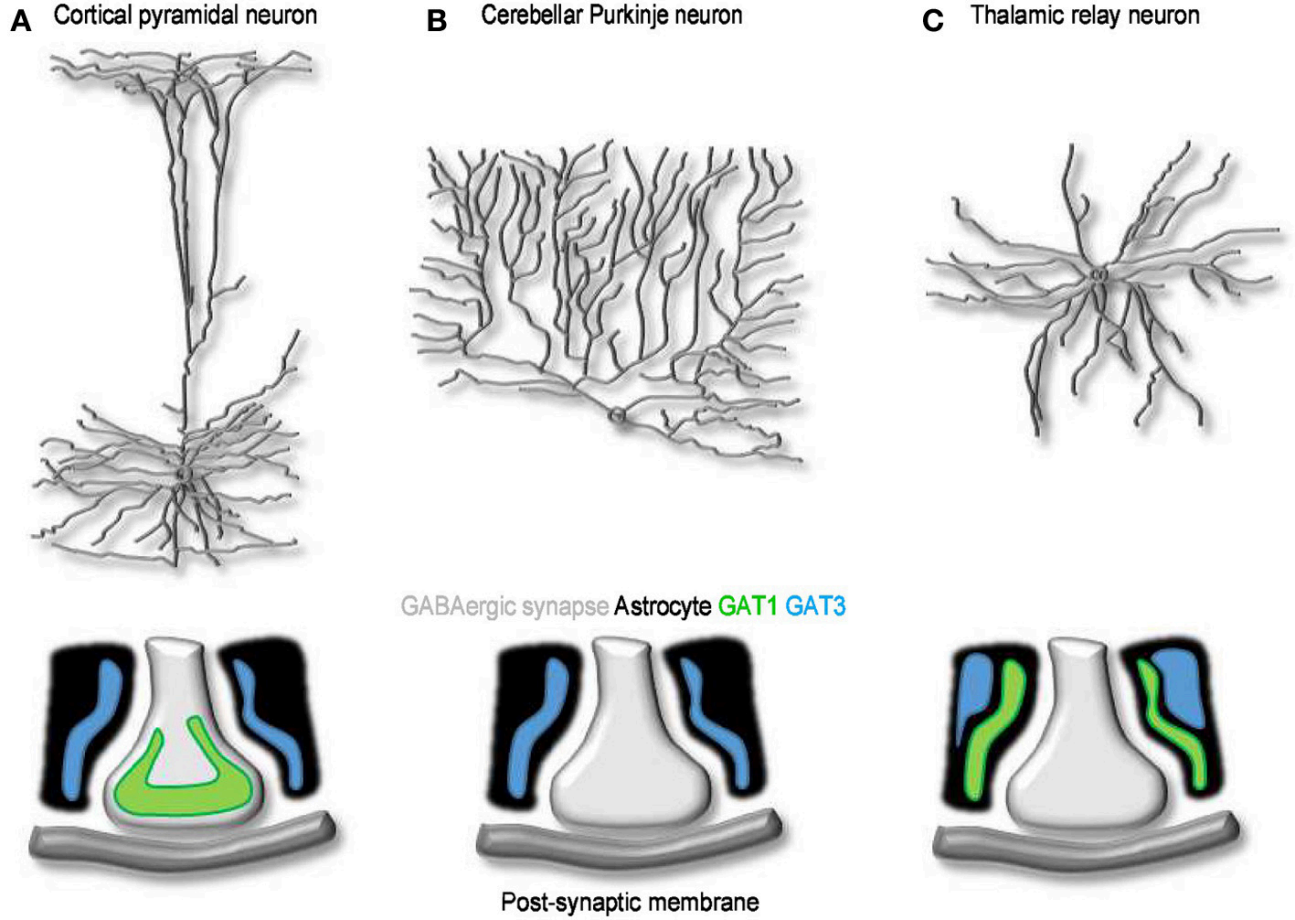
**The cellular and sub-cellular distribution of GABA transporters. (A)** Schematized morphology of cortical pyramidal neuron (top) and of the distribution of GAT1 (green) and GAT3 (blue) at synaptic contacts onto these cells (bottom). **(B,C)** As in A, for Purkinje **(B)** and thalamic relay neurons **(C)**. At GABAergic synapses onto cortical neurons, GAT1 and GAT3 are expressed mainly in pre-synaptic terminals and in neighboring astrocytic processes, respectively. Purkinje neurons lack neuronal GABA transporters; Bergmann glia cells express GAT3. GABA uptake at synaptic contacts onto thalamic relay neurons is mediated by GAT1 and GAT3. Both transporters are located in astrocytes: GAT1 is located closer to the synapse and clears GABA released during phasic events; GAT3 is located further away and regulates the basal, tonic GABA concentration in the extracellular space (Beenhakker and Huguenard, 2010).

At the electron microscope level, the punctate GAT1 immunoreactivity is mainly confined to the axon terminals of symmetrical synapses in the neocortex (Figure 4A). For the most part, this labeling overlaps with that of GAD67-positive terminals located near neuronal cell bodies, axon initial segments and proximal dendrites (Minelli et al., 1995; Ribak et al., 1996), but some GAT1 immunoreactivity has also been detected in distal astrocytic processes (Minelli et al., 1995; Ribak et al., 1996) and post-synaptically, in the dendrites and soma of non GABAergic neurons (Yan et al., 1997). The pre-synaptic neuronal location of GAT1 seems suited for GABA recycling in the pre-synaptic terminal following a release event. This suggests that GABA uptake, not only GABA biosynthesis, is essential to sustain GABAergic synaptic transmission. The function of post-synaptic GAT1 in incompletely understood, but if we could draw an analogy with what we have learnt from the activity of neuronal post-synaptic transporters at excitatory synapses (Scimemi et al., 2009), we would probably infer that they may limit GABA escape from the synaptic cleft toward extra-synaptic territories. The density of expression of GAT1 in cortical and cerebellar plasma membranes has been estimated with fluorescent labeling experiments (Chiu et al., 2002). According to these findings, GAT1 is expressed at a density of 500–800 μm^−2^, 61–63% of the total density of expression of GAT1 in intracellular and plasma membranes (800–1300 μm^−2^). These tentative estimates are useful to develop quantitative frameworks to determine the function of GABA transporters in the synapse (Scimemi, 2014). Because they represent average *density* values, however, they imply that synapses with a large surface area express a higher *number* of GABA transporters than small synapses. There is no clear experimental evidence that supports this assumption. Another important piece of information that is currently missing is whether the relative abundance of GAT1 vs. GAT3, and their relative contribution to synaptic function, varies across synapses with different levels of astrocytic coverage. Therefore, one of the current major limitations is that our understanding of GABA transporters is based on “average” quantitative estimates that do not capture the effect of cell-to-cell variability in GABA transporter expression/activity on synaptic function. There are in fact indications that the expression level of GAT1 vary significantly across GABAergic interneurons. For example, in the hippocampus, GAT1 is more abundantly expressed in *stratum radiatum* rather than *stratum oriens* interneurons (Engel et al., 1998). Accordingly, the amplitude and time course of GABAergic IPSCs evoked in CA1 pyramidal cells by stimulating *stratum radiatum* interneurons show higher sensitivity to the GAT1 inhibitor tiagabine than those evoked by stimulating *stratum oriens* interneurons (Engel et al., 1998). There are examples of GABAergic cells, like cerebellar Purkinje neurons, that lack GAT1 and any designated mechanism for GABA uptake in the pre-synaptic terminal (Minelli et al., 1995). Here the GABA transporter responsible for GABA clearance from the extracellular space is GAT3, which is highly expressed in Bergmann glial cell processes surrounding the synaptic terminal (Figure 4B). Something similar occurs in the thalamus, where GABA transporters are only expressed in astrocytes (De Biasi et al., 1998; Vitellaro-Zuccarello et al., 2003). Interestingly, in the thalamus, GAT1 and GAT3 may occupy distinct domains within the astrocytic membrane, with GAT1 located closer to synaptic contacts than GAT3 (Beenhakker and Huguenard, 2010) (Figure 4C). This peculiar distribution may allow GAT1 and GAT3 to have different roles on synaptic function: the former limiting GABA escape from the synaptic cleft during phasic synaptic transmission, the latter controlling the ambient GABA concentration mediating tonic inhibition (Beenhakker and Huguenard, 2010). These findings are consistent with the recently emerging view that GAT1 and GAT3 regulate different signaling pathways, mediated by GABA released via vesicular and non-vesicular mechanisms (Song et al., 2013), during low-frequency or sustained neuronal activity (Kersante et al., 2013).

## The functional role of GABA transporters

**Figure 5:**
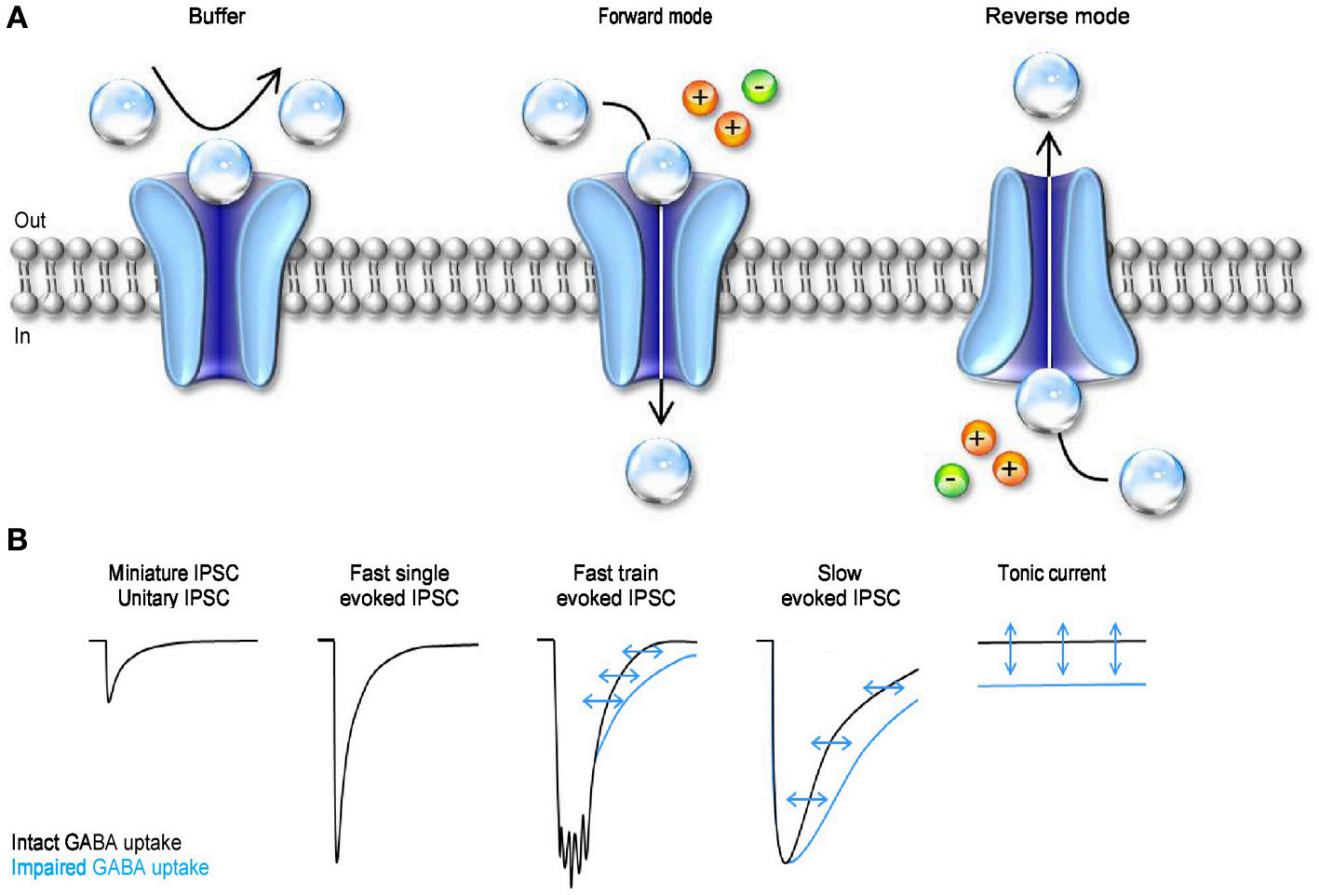
**The mode of action and functional effect of GABA transporters on synaptic transmission. (A)** Schematic representation of the three different modes of action of GABA transporters. GABA molecules (transparent spheres) can be rapidly bound by GABA transporters. Not all the GABA molecules that are bound by the transporters are also translocated across the cell membrane. Under these conditions, the transporters act as buffers (left). GABA uptake is coupled to the movement of Na^+^ (orange sphere) and Cl^−^ (green sphere) across the membrane. In forward mode, GABA transporters remove GABA from the extracellular space (middle). GABA transporters can operate in revered mode (i.e. release GABA in the extracellular space) if the driving force for Na^+^/Cl^−^ favors the movement of these ions outside the cell, and if the intracellular concentration of GABA is sufficiently high to be bound by the transporters (right). **(B)** Schematic representation of the effects of GABA uptake on small, fast, coincident and tonic GABAergic currents. The black traces represent currents recorded in control conditions, with GABA uptake intact. The blue traces represent currents recorded when GABA transporters are blocked.

GABA transporters can transiently bind extracellular GABA, remove it from the extracellular space and, under appropriate ionic conditions, translocate it from the cytosplasm back into the extracellular space (Figure 5A). The diversity of these effects indicates that the functional implications of GABA uptake on synaptic transmission are multiple and complex. There have been apparently conflicting results on the ability of GABA transporters to control the time course of GABAergic currents. This is because inhibiting GABA uptake markedly prolongs the neuronal response to iontophoretic GABA applications (Curtis et al., 1976; Brown and Galvan, 1977; Brown et al., 1980; Alger and Nicoll, 1982; Dingledine and Korn, 1985) and to repetitive synaptic stimulations (Roepstorff and Lambert, 1992; Thompson and Gahwiler, 1992; Isaacson et al., 1993; Draguhn and Heinemann, 1996; Overstreet and Westbrook, 2003) (Figure 5B). It also prolongs the late phase of evoked inhibitory post-synaptic currents [IPSCs; (Dingledine and Korn, 1985; Isaacson et al., 1993)] but has little effect on the amplitude and initial decay of action potential independent miniature and unitary IPSCs (Dingledine and Korn, 1985; Rekling et al., 1990; Thompson and Gahwiler, 1992; Isaacson et al., 1993; Rossi and Hamann, 1998; Overstreet and Westbrook, 2003) (Figure 5B). The discrepancy between these results has been partly resolved over the years based on theoretical and experimental works showing that: (1) the ability of GABA transporters to alter receptor activation depends on the amplitude and time course of the GABA concentration transient; (2) GABA diffusion and GABA receptor kinetics—not GABA uptake—shape the profile of synaptic currents evoked by brief agonist concentrations; (3) GABA uptake regulates GABA receptor activation during simultaneous recruitment of neighboring synapses; (4) GABA uptake limits GABA escape from active synapses (i.e., spillover) and therefore controls the spatial specificity of GABAergic transmission (Dingledine and Korn, 1985; Isaacson et al., 1993; Overstreet et al., 2000, 2003). When GABA uptake is intact, there is a progressive decline in the proportion of time that GABA_A_ receptors spend in the open vs. desensitized state at increasing distances from active release sites (Overstreet et al., 2003). This decline becomes more gradual when GABA transporters are blocked, because a larger fraction of GABA_A_ receptors away from the release site open, rather than desensitize, following GABA release (Overstreet et al., 2003). Taken together, these findings indicate that a key role of GABA transporters in cortical microcircuits is to convert a spatially confined signal into a spatially unrestricted wave of inhibition capable of activating a broad range of pre- and post-synaptic GABA_A_ and GABA_B_ receptors. The likelihood with which these broad range effects occur varies across synapses, depending on the agonist concentration profile and the diffusion properties of the synaptic and peri-synaptic environments. In the hippocampus, GABA release from parvalbumin-expressing (PV) interneurons evokes post-synaptic currents that last <1 ms. In contrast, the currents evoked by GABA release from neurogliaform cells last >100 ms (Hajos et al., 2000; Tamas et al., 2003; Olah et al., 2009; Barberis et al., 2011; Capogna and Pearce, 2011; Chittajallu et al., 2013). Slow GABAergic currents are more susceptible to the activity of GABA transporters, suggesting that slow waves of inhibition, rather than rapid point-to-point communication is mainly modulated by GABA transporters (Szabadics et al., 2007). In hippocampal CA1 pyramidal cells, blocking GABA uptake causes a more pronounced increase in somatic than dendritic currents evoked by exogenous GABA applications (Isaacson et al., 1993). This effect has been attributed to the existence a larger proportion of somatic, rather than dendritic, GABAergic inputs onto these cells. As a consequence, the local concentration of GAT1 (the most abundant GABA transporter in this brain region) is higher at the soma than along the dendrites of CA1 pyramidal cells (Gulyas and Freund, 1996; Miles et al., 1996). Interestingly, many peri-somatic GABAergic inputs onto CA1 pyramidal cells come from PV interneurons (Freund and Buzsaki, 1996; Klausberger and Somogyi, 2008). It is therefore puzzling that there is a high concentration of GAT1 in a sub-cellular compartment where individual GABAergic IPSCs have rapid kinetics and are largely unaffected by blocking GABA uptake. One possible explanation is that peri-somatic GAT1 serves to limit GABA diffusion away from the somatic region during repetitive activation of PV cells and toward the somatic region following the onset of slow waves of dendritic inhibition. If confirmed, this hypothesis may suggest that the main functional role of peri-somatic GABA transporters is to maintain a spatial separation between somatic and dendritic GABAergic signals.

An additional form of GABAergic signaling is the one mediated by tonic activation of GABA receptors (Brickley et al., 1996). These currents are due to the continuous presence of a low sub-micromolar concentration of GABA in the extracellular space (Scimemi et al., 2005; Santhakumar et al., 2006; Wu et al., 2007). That is to say that GABA transporters, despite being able to remove GABA from the extracellular space, do not lower its concentration to levels that prevent it from activating GABA receptors. Tonic GABA currents have been recorded in a variety of cells *in vitro* (Brickley et al., 1996; Rossi and Hamann, 1998; Hamann et al., 2002; Nusser and Mody, 2002; Mitchell and Silver, 2003; Semyanov et al., 2003; Stell et al., 2003; Caraiscos et al., 2004; Scimemi et al., 2005; Wojtowicz et al., 2013) and *in vivo* (Chadderton et al., 2004; Duguid et al., 2012; Kersante et al., 2013; Rovo et al., 2014) (Figure 5B). The functional role of tonic GABA currents is to reduce the neuronal input conductance (Cavelier et al., 2005; Farrant and Nusser, 2005), but the consequences that this has on cell excitability depend on the temporal profile of incoming excitatory inputs (Mitchell and Silver, 2003). The tonic GABA current offsets the cell's output firing rate in response to sustained depolarization and alters the slope of the cell's input-output relationship in response to a random train of excitatory inputs (Mitchell and Silver, 2003). In the cerebellum, it enhances the sensitivity of granule cells to evoked vs. spontaneous sensory stimulation (Duguid et al., 2012) and in the thalamus it controls the onset of neural network oscillations (Rovo et al., 2014). Therefore, by regulating tonic forms of signaling, GABA transporters can exert a powerful control of the accuracy with which information is relayed across cells and brain regions. The electrochemical gradient for Na^+^/Cl^−^ determines the direction of GABA transport, and therefore the ability of GABA transporters to reduce or increase the extracellular GABA concentration. Accordingly, previous reports indicate that GABA transporters can operate in reverse mode (Wu et al., 2007). They do so under experimental conditions that mimic ischemia (Allen et al., 2004), but there is an ongoing debate on whether reverse GABA uptake typically occurs under more physiological conditions (Heja et al., 2012; Wojtowicz et al., 2013).

## The regulation of GABA transporter density of expression

**Figure 6:**
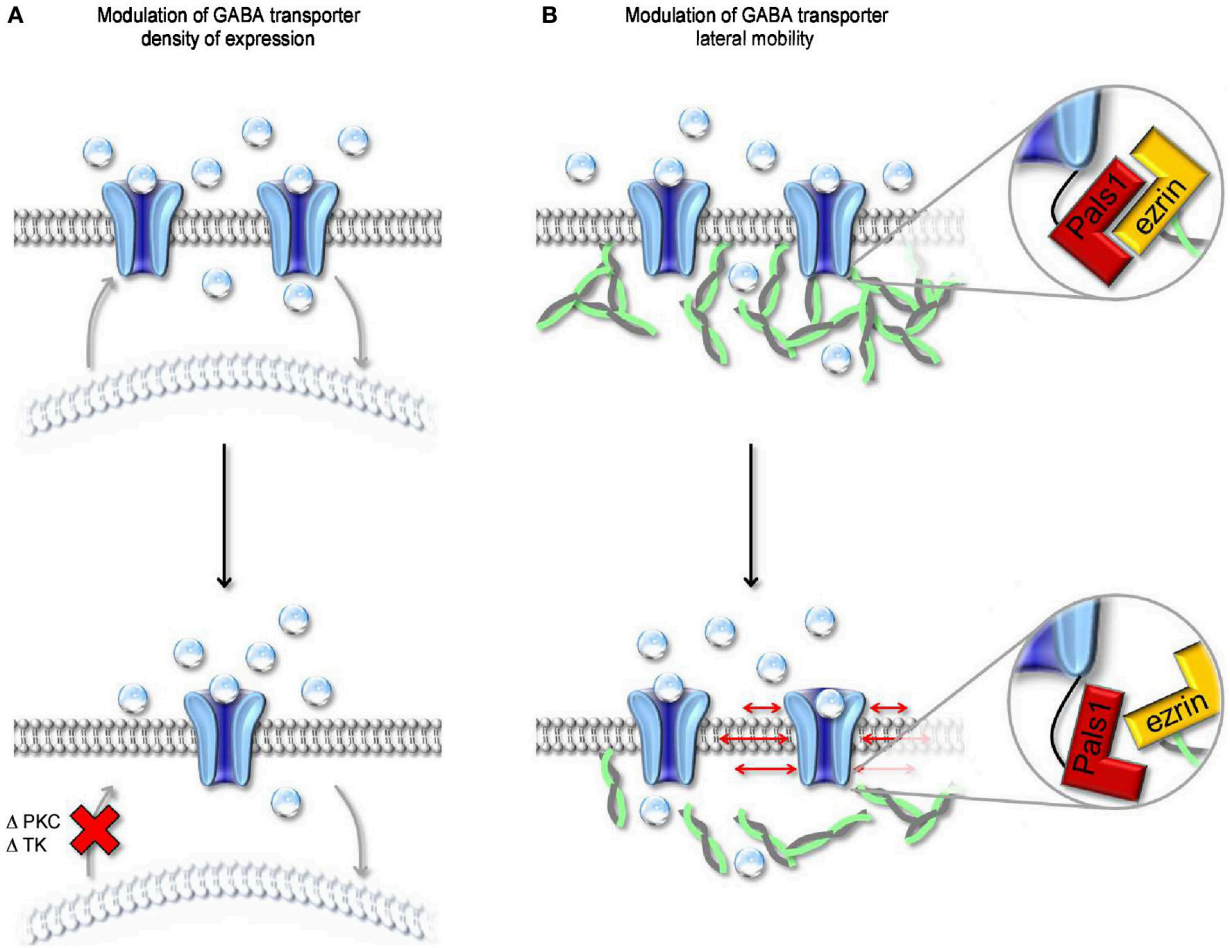
**Modulation of surface expression and lateral mobility of GABA transporters. (A)** Schematic representation of changes in cell surface expression of GABA transporters evoked by altering the activity of PKC and/or tyrosine kinase (TK). Both kinases regulate the trafficking of GABA transporters from intracellular organelles (light, curved lipid bilayer) to the cell membrane (dark, flat lipid bilayer). A reduction in the rate of cell membrane insertion (bottom) leads to a decrease in the cell surface expression of GABA transporters. **(B)** Schematic representation of the molecular interactions mediating anchoring of GABA transporters to the actin cytoskeleton. The MAGUK protein Pals1 mediates the interaction between the C-terminal of GABA transporters and ezrin, an adaptor protein that interacts with actin. Disruption of the interaction between Pals1 and ezrin leads to an increased lateral mobility of GABA transporters in the cell membrane.

GABA transporters, like other neurotransmitter transporters, are dynamically regulated at the level of their surface-to-cytoplasm partitioning and anchoring to the cell membrane (Figure 6). In *Xenopus* oocytes, activating protein kinase C (PKC) with phorbol esters like phorbol-12-myristate-13-acetate (PMA) and 1-oleoyl-2-acetyl-sn-glycerol (OAG), or with *N*-heptyl-5-chloro-1-naphtalenesulphonamide (SC-10) and (-)-indolactam V (indoV) enhances GABA uptake via GAT1, whereas inhibiting PKC with bisindolyilmaleimide reduces it (Corey et al., 1994). Consistent with these findings, inhibiting the phosphatase PP2B (calcineurin) enhances GABA uptake (Corey et al., 1994). Notably, the effects of PKC and PP2B on GABA uptake are entirely post-translational because they can be detected even in the presence of cycloheximide, a protein synthesis inhibitor. PKC and PP2B are thought to alter the trafficking of GAT1 from trans-Golgi and/or low density cytoplasmic vesicles to the plasma membrane by modulating the phosphorylation state of non-consensus sites on GAT1 and/or the activity of second messenger signaling cascades. These changes in the surface-to-cytoplasm expression of GAT1 are associated with changes in the translocation rate of GABA via GAT1 (*V_max_*), not in the Michaelis-Menten constant for GABA uptake via GAT1 (*K_m_*), suggesting that they do not alter GAT1 binding affinity for the substrate (Corey et al., 1994). The magnitude of these effects depends on the abundance of expression of GAT1 in the plasma membrane: the more GAT1 is present on the plasma membrane, the smaller is the modulatory effect of PKC. However, any attempt to generalize the findings obtained in *Xenopus* oocytes to other cell types should be pursued with caution. In rat brain cortical cultures, pharmacological activating PKC with PMA, phospholipase C (PLC) or OAG (the endogenous, membrane permeable analog of DAG generated from phosphatidylinositol breakdown by PLC) does not alter GABA uptake in neurons but decreases it in glial cells (Gomeza et al., 1991). In rat hippocampal cultures, PMA reduces GABA uptake in neurons but not in glial cells (Beckman et al., 1998). Enhancing PKC activity indirectly, with agonists of G-protein-coupled neurotransmitter receptors that lead to PKC activation also reduces GABA uptake in neurons (Beckman et al., 1999). Accordingly, agonists of acetylcholine muscarinic receptors (M1, M3 and M5), glutamate metabotropic receptors (group I) and serotonin receptors (5-HT2) all reduce GABA transporter cell membrane expression by activating PKC (Beckman et al., 1999). Although puzzling and largely unresolved, the discrepancy in the effect of PKC activation on GABA transporters in *Xenopus* oocytes, cultured neurons and glial cells may be attributed to differences in the concentration of PKC in all these different cell types. This suggests that *in vivo* the influence of PKC on GAT1 activity may vary significantly depending on the amount and specific PKC isoform expressed in different neurons and, possibly, in different sub-cellular compartments (Takai et al., 1977; Nishizuka, 1988).

A distinct mechanism through which neurons can regulate the cell-surface expression of GAT1 is via tyrosine kinase phosphorylation of intracellular tyrosine residues (Law et al., 2000) (Figure 6A). These changes in the cell-to-cytoplasm partitioning of GAT1 are due to reduced internalization of the transporter during tyrosine kinase activation (Whitworth and Quick, 2001). In rat hippocampal neurons, the effect of tyrosine kinases activation is similar to that evoked by PKC inhibition, namely an increase in GABA uptake due to enhanced cell membrane expression of GAT1. Under physiological conditions, a possible candidate trigger for tyrosine kinases activation is the growth factor BDNF, which is synthesized and secreted by pyramidal neurons but has target receptors in various types of GABAergic interneurons (Ernfors et al., 1990). During development, GABA-induced membrane depolarization leads to opening of L-type voltage-gated calcium channels and release of BDNF, which in turn promotes interneuron differentiation (Marty et al., 1996a,b). By increasing GABA uptake, BDNF may alter the concentration of GABA in pre-synaptic terminals and its lifetime in the extracellular space. As GABA becomes hyperpolarizing (Ben-Ari et al., 1989), it loses its ability to activate L-type calcium channels and trigger BDNF synthesis and release (Berninger et al., 1995). The consequent reduction in GABA uptake may contribute to the reduced neurotrophic effects of GABA in the mature brain.

## The regulation of GABA transporter lateral mobility

Previous works indicate that the localization of individual GAT1 molecules within the plasma membrane is not fixed in time and is not entirely predicted by passive diffusion in the lipid bilayer (Imoukhuede et al., 2009). This is in agreement with the results of FRAP experiments in neuroblastoma 2a cells, in which 50% of the total pool of GAT1 present in the cell membrane is mobile while the remaining 50% is immobile. Within the immobile population, a fraction of GAT1 molecules is indirectly tethered to the actin cytoskeleton via a PDZ-interaction between the C-terminal domain of GAT1, the MAGUK protein Pals1 (Mchugh et al., 2004) and ezrin, an adaptor protein that connects Pals1 to actin (Imoukhuede et al., 2009) (Figure 6B). Disrupting the interaction between GAT1 and the actin cytoskeleton (i.e., rendering all GAT1 molecules mobile) has been shown to increase GABA uptake (Imoukhuede et al., 2009). However, these conclusions are based on experimental measures of radioactive tracer flux typically obtained under steady-state conditions (i.e., in the presence of an unknown, but constant agonist concentration). There is hardly anything that is at steady-state during synaptic transmission. For example, the GABA concentration varies dramatically with time and distance from the release site. It is therefore challenging to get a simple and accurate intuition of whether and how changes in the lateral mobility of GABA transporters can affect the GABA concentration profile inside and outside the synaptic cleft. A simple modeling approach suggests that increasing the mobility of GABA transporters along the cell membrane does not alter the lifetime of GABA in the extracellular space but allows it to diffuse further away from an active release site (Scimemi, 2014). This suggests that intracellular signaling cascades that regulate the surface expression and mobility of GABA transporters may ultimately control the spatial specificity of GABAergic transmission in the brain.

## Conclusions

There have been tremendous advances in our understanding of the molecular architecture and function of GABA transporters. A number of works has shown that there are intracellular signaling cascades that control the cell surface expression and mobility of GABA transporters, suggesting that the regulation of GABA transporter activity may be more complex than previously thought. It remains currently unclear how these short- and long-term changes in the expression, mobility and activity of GABA transporters affect the time course and spatial spread of GABAergic signals in the brain. This missing piece of information hold the promise of providing novel insights into the regulation of synaptic transmission by GABA transporters and will shed new light on our current understanding of the synaptic mechanisms underlying the generation and maintenance of neuronal network oscillations in distinct regions of the living brain.

### Conflict of interest statement

The author declares that the research was conducted in the absence of any commercial or financial relationships that could be construed as a potential conflict of interest.
